# U1snRNP-mediated suppression of polyadenylation in conjunction with the RNA structure controls poly (A) site selection in foamy viruses

**DOI:** 10.1186/1742-4690-10-55

**Published:** 2013-05-29

**Authors:** Eva-Maria Schrom, Rebecca Moschall, Maximilian J Hartl, Helena Weitner, David Fecher, Jörg Langemeier, Jens Bohne, Birgitta M Wöhrl, Jochen Bodem

**Affiliations:** 1 Institute of Virology and Immunobiology, University of Würzburg, Versbacher Street 7, 97078 Würzburg, Germany; 2 Lehrstuhl Biopolymere, University of Bayreuth, Bayreuth, Germany; 3 Institute of Virology, Hannover Medical School, Hannover, Germany

**Keywords:** Polyadenylation, Foamy viruses, RNA structure, Major splice donor

## Abstract

**Background:**

During reverse transcription, retroviruses duplicate the long terminal repeats (LTRs). These identical LTRs carry both promoter regions and functional polyadenylation sites. To express full-length transcripts, retroviruses have to suppress polyadenylation in the 5′LTR and activate polyadenylation in the 3′LTR. Foamy viruses have a unique LTR structure with respect to the location of the major splice donor (MSD), which is located upstream of the polyadenylation signal.

**Results:**

Here, we describe the mechanisms of foamy viruses regulating polyadenylation. We show that binding of the U1 small nuclear ribonucleoprotein (U1snRNP) to the MSD suppresses polyadenylation at the 5′LTR. In contrast, polyadenylation at the 3′LTR is achieved by adoption of a different RNA structure at the MSD region, which blocks U1snRNP binding and furthers RNA cleavage and subsequent polyadenylation.

**Conclusion:**

Recently, it was shown that U1snRNP is able to suppress the usage of intronic cryptic polyadenylation sites in the cellular genome. Foamy viruses take advantage of this surveillance mechanism to suppress premature polyadenylation at the 5’end of their RNA. At the 3’end, Foamy viruses use a secondary structure to presumably block access of U1snRNP and thereby activate polyadenylation at the end of the genome. Our data reveal a contribution of U1snRNP to cellular polyadenylation site selection and to the regulation of gene expression.

## Background

Most cellular mRNAs are polyadenylated. Polyadenylation (poly(A)) is provided, by four sequence elements: the polyadenylation signal (poly(A) signal), the cleavage site (poly(A) site), G/U-rich downstream elements (DSE), and upstream cleavage factor I binding sites (for review see [1-4]). The polyadenylation reaction can be characterized as a two-step process: 1) RNA is cleaved at the polyadenylation site, and 2) the poly(A) tail is added. Retroviruses use novel mechanisms to control polyadenylation and thus serve as useful tools to study regulation of this process [5]. The retroviral genome is flanked by two long terminal repeats (LTRs) with identical sequences but different functions [6,7]. After integration of the viral genome into the cellular DNA, the 5′LTR serves as a promoter for viral transcription, and polyadenylation of the viral transcripts occurs at the 3′LTR. The LTRs consist of three distinct regions: U3, which harbours the promoter; R, which possesses the transcriptional start site at its 5′end; and U5, which begins with the poly(A) site [6,7]. Retroviruses must suppress transcript RNA cleavage and subsequent polyadenylation in the 5′LTR, but activate 3′end processing in the 3′LTR [5]. An active poly(A) site in the 5′LTR would cause a premature cleavage of viral RNAs and impair viral gene expression. If only the cleavage step was suppressed, full-length genomic preRNAs would be produced, while suppression of poly(A) addition would result in suppression of full-length transcripts. The regulation of retroviral polyadenylation appears to depend on cellular factors and on viral RNA sequences, as no retroviruses have been shown to encode proteins that impact polyadenylation. To date, three different types of retroviral mechanisms for polyadenylation regulation have been identified (for review see [5]).

**Figure 1:**
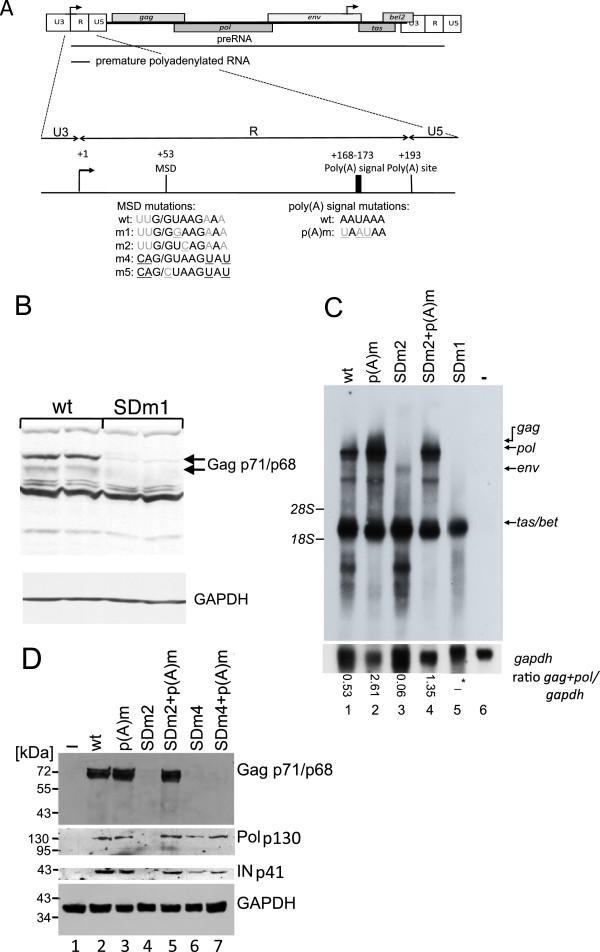
**The MSD is required for*gag,pol,*and*env*expression.** (**A**) Structure of the FV R region. Mutations of the MSD and poly(A) signal are depicted below the scheme. MSD nucleotides complementary to the U1snRNA are in black, and mutations are underlined. Arrows indicate the positions of the promoters. (**B**) Western blotting analysis of BHK-21 cells transfected with pHSRV2 or pHSRV2-SDm1. Transfections were performed in duplicate. Gag was detected with a Gag-antiserum, and GAPDH served as a loading control. (**C**) Northern blotting analysis of total RNA from BHK-21 cells transfected with pHSRV-13 MSD derivatives showing that the MSD is required for expression of 5’LTR derived transcripts (compare lanes 1 with lanes 3 and 5) and that an additional poly(A) inactivation rescues the SDm2 phenotype. A *tas*/*bet*-specific probe was used to detect viral RNAs. The positions of the 18S (1.9 kb) and 28S (4.7 kb) rRNAs are indicated. Ratios of gag+pol/gaph transcripts are indicated below the blots. In case of pHSRV13-SDm1 (−*), the ratio could not be determined due to the lack of *gag* and *pol* transcripts. (**D**) Western blotting analysis of BHK-21 cells using a monoclonal Gag antibody or a polyclonal integrase-specific antiserum. The latter detects the unprocessed Pol precursor (p130) as well as integrase (p41). GAPDH served as a loading control.

The first such mechanism involves polyadenylation signals encoded upstream of the promoter start site. This simple and obvious type of regulation has been described for Rous sarcoma virus (RSV), mouse mammary tumour virus (MMTV), and human T-lymphotropic virus type 1 (HTLV-1). In these viruses, the essential poly(A) signals are localized in the U3 region [8-10]. Since transcription starts within the R region at the 5′LTR the U3 region will only be transcribed at the 3′end of the RNA (Figure 1A). Hence, suppression of the 5′LTR poly(A) site is not required [8], since the essential polyadenylation signal is not present at the 5′RNA end. These viruses support polyadenylation only at the 3′LTR. As a consequence of the simultaneous recognition of the poly(A) signal and the DSE, located in U5, viruses, which encode the poly(A) signal in U3, require a short R region. Otherwise concurrent binding of the polyadenylation complex would be prevented by the distance between the poly(A) signal and the DSE [9]. On the other hand, the R region of HTLV-1 encompasses 228 nucleotides [6,11], which would prevent polyadenylation. However, HTLV-1 encodes an RNA element with extensive secondary structure named Rex responsive element, which is used to bridge this gap [8].

A second mechanism for suppression of polyadenylation involves elements downstream of the LTR. Splicing and polyadenylation occur co-transcriptionally and are coupled processes (for review see [1,2]). It has been reported that splicing can inhibit or enhance polyadenylation [1,12-15]. In human immunodeficiency virus type 1 (HIV-1), polyadenylation at the 5′LTR is suppressed by the major splice donor (MSD), located 195 nucleotides downstream of the poly(A) signal. [16-18]. The current model suggests that binding of the U1 70k protein, which is part of U1snRNP, inhibits 3′end processing at 5′LTR [19,20]. Furthermore, polyadenylation efficiency was shown to be dependent on the distance between the MSD and the polyadenylation signal. The poly(A) site in the 3′LTR is activated, because the MSD is not present at the 3′end of the RNA. In addition, it has been demonstrated that signals in the HIV-1 U3 region enhance polyadenylation [21,22].

The third mechanism for polyadenylation suppression involves weak polyadenylation sites in both LTRs. The Moloney Murine Leukaemia Virus (MoMLV) harbours a weak poly(A) site, and sequences in the R region are required for poly(A) site regulation [9]. In contrast to HIV-1, the control of polyadenylation in the 5′LTR of MoMLV is MSD-independent, although the positioning of the MSD and the poly(A) site is similar in all orthoretroviral genomes. However, the MLV MSD was shown to be inefficiently recognized by U1snRNP due to a combination of RNA secondary structure and low complementarity to U1snRNA [23]. The disadvantage of this mechanism is the accidental premature polyadenylation of viral transcripts, which results in less efficient expression of viral genomic RNA and all viral genes. However, it enables some retroviruses to capture cellular proto-oncogenes by read-through at the 3′end of the 3′LTR.

Gene expression of foamy viruses (FVs) differs from that of orthoretroviruses [24]. Two promoters, one located in the LTR and the other in the *env* region, initiate gene expression. The activity of both promoters is dependent on the viral transactivator protein Tas. FV *pol* is encoded by a specific spliced transcript. Furthermore, the structure of the FV R region is unique (Figure 1A) [25]. The prototype FV (PFV) R region is 193 nucleotides in length (Additional file 1). The MSD is located in the R region [26]. Despite relatively low complementarity, bioinformatics analysis predicts that the MSD constitutes a strong 5′splice site [27,28]. Unfortunately, experiments to determine the MSD strength have not been performed so far. The MSD is, for unknown reasons, important for *gag* and *pol* expression [29,30]. The poly(A) signal is located at +168 to +173 and followed by the poly(A) site at +193 [26] (Figure 1A and Additional file 1). Thus, both the 5′ and 3′ends of the viral RNA contain all signals thought to be required for polyadenylation. However, polyadenylation cannot be regulated as in RSV and MMTV, since the polyadenylation signal is localized in the R region and not in U3. In addition, compared with their orthoretroviral and endogenous retroviral counterparts, FVs have the opposite configuration of the MSD and poly(A) site, indicating that the regulation of polyadenylation might differ from that of HIV-1. On the other hand, it has been shown that the FV LTR suppresses read-through efficiently [31], implying that FVs encode a strong poly(A) site. This would exclude the mechanism described for MoMLV. These facts make FVs an excellent model system to study the requirements for both repression and activation of polyadenylation.

In this work, we analysed how identical nucleotide sequences in both FV LTRs can execute opposite functions. We provide insights into the regulation of polyadenylation and show that the RNA structure affects splice site recognition.

## Results

### The major splice donor in the 5’LTR is required for FV *gag* expression

FVs possess a poly(A) signal and site in each LTR. Consequently, both repression of the poly(A) signal in the 5′LTR and promotion of polyadenylation in the 3′LTR are required to express full-length 5′LTR-derived transcripts. Surprisingly, previous experiments showed that inactivation of the MSD by site-directed mutagenesis resulted in complete loss of *gag* expression ([29,32] Löchelt and Bodem, unpublished observation). To confirm these results, we transfected baby hamster kidney (BHK-21) cells with either the proviral pHSRV2 plasmid or the 5′LTR MSD mutant clone pHSRV2-SDm1 [32]. The latter carries a single nucleotide exchange in the MSD (Figure 1A, SDm1). This leaves 6 nucleotides complementary to the cellular U1snRNA but disrupts the continuous binding site of 5 nucleotides in the MSD (Figure 1A). Two days after transfection, cells were harvested, and *gag* expression was analysed by Western blotting (Figure 1B). The SDm1 mutation was inserted into the proviral pHSRV13 backbone [33] for cloning reasons, and all other proviral constructs of this study were based on pHSRV13, too. As Gag protein levels were undetectable in cells transfected with the pHRSV13-SDm1 construct (Figure 1B), we used Northern blotting to analyse expression of *gag*-encoding genomic RNAs (size 11 kb). In cells transfected with the pHSRV13-SDm1 plasmid, neither *gag*-encoding genomic RNA nor *pol* or *env* RNA was retrieved (Figure 1C, lane 5), indicating that the mutation in SDm1 might activate cleavage and polyadenylation similar to inactivation of the HIV-1 MSD [16,18]. Signals below p68 are due to an unspecific reactivity of the serum.

To further investigate the MSD mutant phenotype, we introduced a different single nucleotide mutation (SDm2) into the MSD at the 5′LTR (Figure 1A). SDm2 also encodes 6 nucleotides complementary to U1snRNA (Figure 1A). No LTR-derived transcripts were observed in cells transfected with pHSRV13-SDm2 (Figure 1C, lane 3). To correlate this effect to polyadenylation, we mutated the poly(A) signal in the 5′LTR (wild-type, AAUAAA; p(A)m, UAAUAA) in the wild-type and the SD2 mutant (SDm2+p(A)m). This inactivation of the poly(A) signal should restore expression only if polyadenylation was activated by the MSD mutation (Figure 1A). Transfection of cells with plasmids containing p(A)m resulted in increased expression of LTR-derived transcripts (Figure 1C, lane 2), indicating that some of the transcripts were already polyadenylated at the poly(A) site in the wild type 5′LTR. The inactivation of the poly(A) signal in the 5′LTR in the pHRSV13-SDm2+p(A)m plasmid restored expression of LTR transcripts to wild-type levels, indicating that the pHRSV13-SDm2 mutation might have activated polyadenylation at the 5′LTR (Figure 1C, lane 4). The *tas*/*bet* expression was similar in all isolated RNAs, as both genes are expressed from the internal promoter. To analyse influences of a strong MSD, an additional MSD mutant (SDm4), encoding 11 nucleotides complementary to U1snRNA, was generated as well (Figure 1A). In cells transfected with pHSRV13-SDm2, Gag, the Pol precursor and integrase were undetectable by Western blotting, but expression of these proteins was restored by the additional inactivation of the poly(A) signal (Figure 1D, lanes 4 and 5). Cells transfected with pHSRV13-SDm4 expressed *pol*, but Gag was undetectable (Figure 1D, lane 6). The additional inactivation of the poly(A) signal did not restore Gag expression, which could be assigned to enhanced splicing (data not shown). These results show that the MSD is required for expression of LTR-derived transcripts. In addition, the results with the poly(A) signal mutants support the hypothesis that the MSD is essential for suppression of polyadenylation or RNA cleavage at the 5′LTR.

### Mutations in the MSD of the 5’LTR lead to premature cleavage

**Figure 2:**
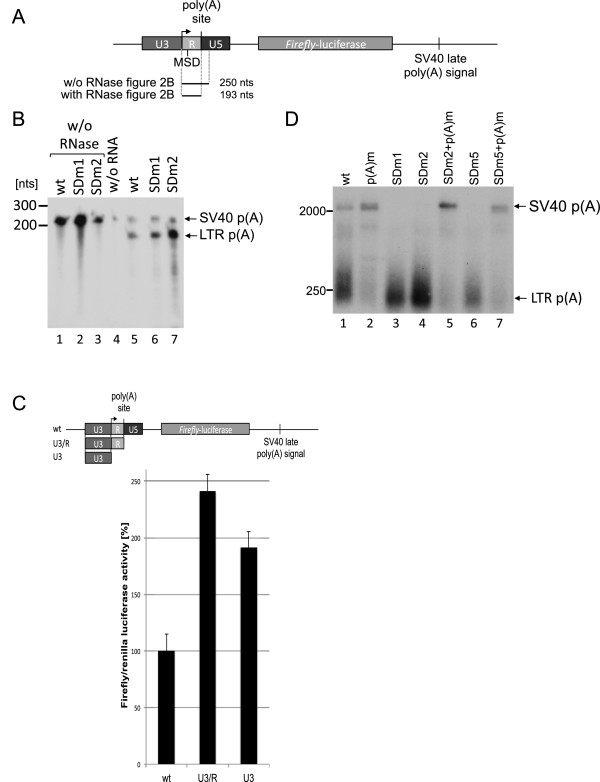
**Mutation of the MSD activates polyadenylation in the 5’LTR.** (**A**) Overview of the luciferase constructs and the position of the RPA probe (**B**) RPA of transcripts expressed by wild-type, SDm2, and SDm1 constructs (above the panel). Probes without RNase digestion (lanes 1–3) and the digest without cellular RNA (lane 4) were used as controls. The localization of the probes is indicated in the plasmid schema above the panel. (**C**) Luciferase assays of the U3, U3R, and LTR promoter activity showing that sequences in R and U5 act negatively on gene expression. The constructs are depicted above the panel. Bars represent the mean value of three independent transfections, and the error bars represent the standard deviation. (**D**) Southern blot of RT-PCR products using oligo d(T) as primer for cDNA synthesis and +1 and oligo d(T) as primers for PCR. The wild-type and SDm2-derived short transcripts are polyadenylated at the 5′LTR. Positions of size markers and plasmids used for transfection are indicated. PCR products were hybridized to an antisense RNA probe encompassing nucleotides +1 to +250.

In order to analyse repression of polyadenylation at the 5′LTR in a quantitative way and to exclude influences of the 3′LTR, we constructed reporter plasmids encompassing the complete pHSRV13 5´LTR encoding either the wild-type MSD or the SDm1 or SDm2 mutants in the pGL3 vector backbone. Thus, the U3 promoter drives firefly luciferase expression (Figure 2A). The resulting construct possesses two poly(A) sites, one in the 5´LTR and a second SV40-derived poly(A) signal 3′ of the luciferase gene. If cleavage at the LTR poly(A) site is suppressed, firefly luciferase should be expressed (Figure 2A and Additional file 1: Figure S1). On the other hand, if the LTR poly(A) signal is active, the RNA should be cleaved at the LTR poly(A) site, and luciferase expression should be impeded (Additional file 1: Figure S1).

These constructs were used to analyse the SDm1 and SDm2 LTR variants by ribonuclease protection assays (RPAs) (Figure 2B). For the RPA, three antisense RNA probes complementary to nucleotides +1 to +250 – encoding the wild-type, the SDm1, or the SDm2 MSD – were produced. A specific probe for each construct was necessary to avoid cleavage of the RNA probe at the mutated MSD due to non-pairing. All transfections included a Tas expression plasmid (pCMVTas) as expression of the viral transactivator Tas is required to activate the LTR promoter. Transcripts cleaved/polyadenylated at the LTR poly(A) site should result in a protected 193-nucleotide fragment (Figure 2A), whereas suppression of this site should result in a 250-nucleotide fragment. The RPAs showed that suppression of the poly(A) site at the 5′LTR is incomplete and that suppression of polyadenylation acts at the first step of polyadenylation, i.e. RNA cleavage is inhibited. The majority of all transcripts were cleaved at the LTR poly(A) site (Figure 2B). Reporters carrying SDm1 (pGL3SDm1) or SDm2 (pGL3SDm2) showed strong increases in RNAs cleaved at the LTR poly(A) site compared to the wild-type (Figure 2B). This experiment indicates that 1) about 40% of all transcripts are prematurely cleaved in the wild-type context, and 2) the SDm1 and SDm2 mutations result in a further increase in transcripts cleaved at the LTR polyadenylation site, confirming that the MSD indeed suppresses RNA cleavage.

To analyse the impact of the essential G/U-rich DSE in the U5 region on incomplete suppression of polyadenylation we cloned either the U3- or the U3R-promoter regions in the pGL3 backbone. In this set of experiments, a CMV-promoter-driven *Renilla* luciferase expression plasmid was co-transfected to allow normalization of transfection efficiencies. Two days after transfection, cellular lysates were prepared, and both firefly and *Renilla* luciferase activities were measured (Figure 2C). The deletion of the U5 region (Figure 2C, second bar (U3R)), which includes the deletion of the DSE required for polyadenylation, resulted in an approximately 2.5-fold increase in the luciferase activity, whereas a plasmid encoding only the U3 region exhibited an approximately 2-fold increase (Figure 2C, third bar (U3)). These findings, along with the increase of the genomic transcript with the SDm2+p(A)m double mutant (Figure 1C), support the view that suppression of the FV polyadenylation at the 5′LTR is incomplete and that the U5 region indeed contains a DSE.

To show that the short transcripts are not only cleaved but also polyadenylated at the 5´LTR, an oligo d(T) primed RT-PCR was performed with RNA of cells transfected with the reporter plasmids (Additional file 1). FV cDNAs were amplified with oligo d(T) and the +1 primer. The PCR products were blotted and hybridised to an antisense RNA probe complementary to nucleotides +250 to +1 to verify the FV origin of the PCR products. This analysis revealed strong amplicons of transcripts polyadenylated at the 5´LTR from cells transfected with the wild-type, the SDm1 or SDm2 mutants (Figure 2D, lanes 1, 3, and 4) showing an almost complete polyadenylation at the LTR for both MSD mutants (Figure 2D, lanes 3 and 4). The inactivation of the poly(A) signal resulted in the loss of RNA species polyadenylated at the 5´LTR (Figure 2D, lanes 2, 5 and 7). This shows that the short transcripts are indeed both cleaved and polyadenylated. Furthermore, it supports our hypothesis that the suppression of polyadenylation in the wild-type LTR is incomplete and is regulated via the MSD, possibly by U1snRNP interaction.

### Binding of U1snRNP is required for suppression of polyadenylation

**Figure 3:**
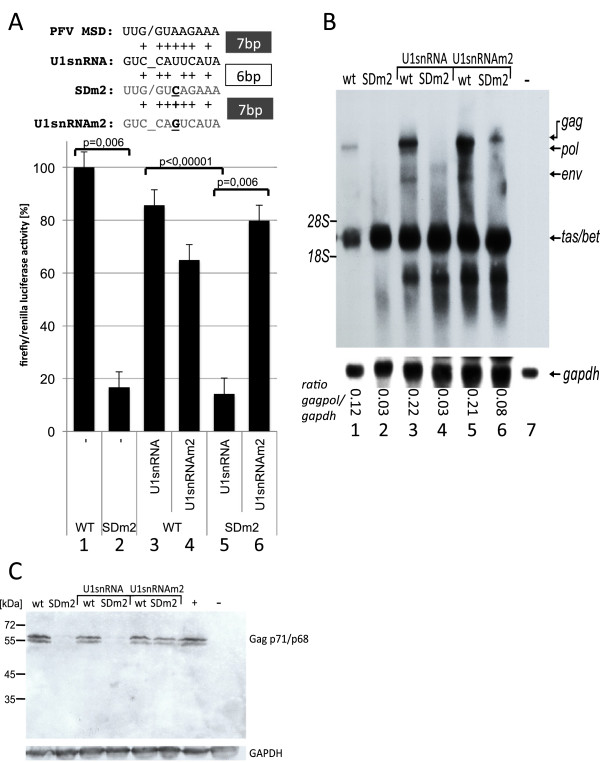
**The occlusion of polyadenylation is U1snRNA**-**dependent.** (**A**) Luciferase activity in BHK-21 cells. Co-transfection with the pGL3LTR derivatives and either wild-type or U1snRNAm2 expression construct complementary to SDm2. The latter restored suppression of polyadenylation in cells transfected with SDm2. An alignment of U1snRNA and splice donor RNA sequences is shown above the diagram (mutated nucleotides are shown in bold and underlined). Bars represent the mean value of three independent transfections, and the error bars represent the standard deviation. The significance of the reduction by the SDm2 mutation or increase by co-transfection of U1snRNAm2 was calculated by the paired two-sample t-test. p-values are indicated. (**B**) The SDm2 mutant is rescued by U1snRNAm2 co-transfection in a proviral context. Northern blotting analysis of RNA from BHK-21 cells co-transfected with either pHSRV13 or pHSRV13SDm2, and wild-type or SDm2 U1snRNA expression constructs. Viral RNAs were visualised using a *tas*-specific probe. The positions of the 18S (1.9 kb) and 28S (4.7 kb) rRNAs are indicated. The normalised amounts of gag/pol transcripts are depicted below the lanes. (**C**) Gag, Tas, and GAPDH were analysed by Western blotting. PFV-infected BHK-21 cells (+) and untransfected cells (−) served as controls.

To show that U1snRNP binding to the MSD regulates poly(A) suppression, we performed experiments with a mutated U1snRNA that was complementary to 7 nucleotides of SDm2 (Figure 3A). Expression of this U1snRNA mutant should restore suppression of polyadenylation only if snRNP binding is a determinant for suppression of polyadenylation. Cells were co-transfected with a plasmid encoding the wild-type U1snRNA or the mutant U1snRNA (U1snRNAm2) and with the luciferase reporter constructs (Figure 3A). A CMV-promoter-driven *Renilla* luciferase expression plasmid was co-transfected to allow normalization of transfection efficiencies. Both firefly and *Renilla* luciferase activities were measured (Figure 3A). The reporter carrying the pGL3SDm2 mutant showed strongly reduced luciferase activity compared to the wild-type LTR construct, similar to the reduction observed in the RPA (Figure 3A). As described before [34], we observed that over-expression of the wild-type U1snRNA lowered the luciferase expression of the wild-type pGL3LTR significantly (p = 0.006) (Figure 3A, compare bars 1 and 3), indicating that U1snRNA over-expression exerts some side effects. However, the ratio of luciferase activity seen with the pGL3LTR wild-type and the SDm2 reporter with and without co-transfection of the wild-type U1snRNA remained unchanged (Figure 3A, compare reduction from bar 1 to 2 (p = 0.006) and from bar 3 to 5 (p<0.00001)). Co-transfection of the U1snRNAm2 construct strongly increased expression of the SDm2 construct (Figure 3A, compare bars 5 and 6), showing that U1snRNA binding can reverse the impact of the SDm2 mutation. This result supports the hypothesis that U1snRNA binding is required for suppression of transcript cleavage and subsequent polyadenylation.

To analyse whether expression of 5´LTR-derived transcripts could be restored by U1snRNAm2 expression in the context of proviral MSD mutant constructs, BHK-21 cells were co-transfected with the proviral clones pHSRV13 or pHSRV13-SDm2 and the U1snRNA or U1snRNAm2 expression constructs. We co-transfected a Tas-encoding plasmid to compensate for splicing defects, which might effect Tas expression. The foamy viral transcripts were visualized by Northern blotting using a *tas*-specific probe (Figure 3B). Co-expression of U1snRNA or the mutated U1snRNA did not influence the ratio of 5´LTR-derived transcripts of pHSRV13 (Figure 3B, lanes 3 and 5). In contrast, co-transfection with the U1snRNAm2 construct enhanced the LTR-promoter-derived *gag* expression of pHSRV13-SDm2, as seen in the luciferase model. To further verify these data, quantities of Gag expression were analysed by Western blotting with a Gag-specific monoclonal antibody (Figure 3C). The pHSRV13-SDm2 mutant did not express a significant amount of Gag. The Gag expression levels of pHRSV13 and its SDm2 mutant were not affected by over-expression of the wild-type U1snRNA, but expression of U1snRNAm2 restored Gag expression of pHSRV13-SDm2 to wild-type levels (Figure 3C).

The experiments with the proviral plasmids gave rise to similar results on the RNA and protein levels and show that U1snRNA is required for the expression of LTR-derived transcripts. Furthermore, the results correlate well with the quantitative data obtained with the luciferase-reporter-based model system. The higher sensitivity of the reporter system allowed us to detect effects of the mutated U1snRNA on the wild-type MSD that could not be visualized by Western or Northern blotting.

### Suppression of the poly(A) site is independent of splicing

**Figure 4:**
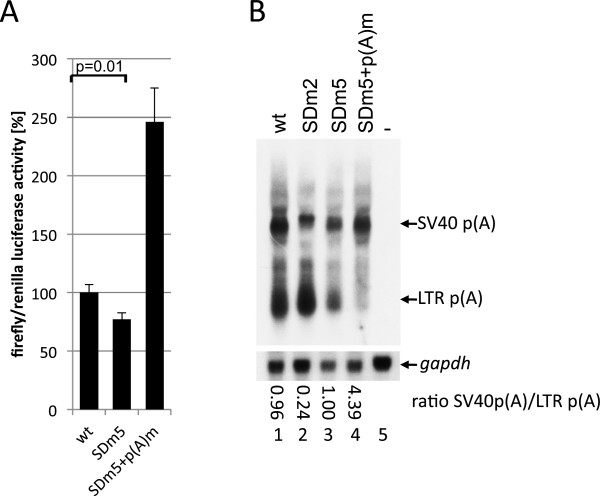
**Regulation of polyadenylation is independent of splicing.** (**A**) Luciferase assay of BHK-21 cells transfected with pGL3 derivatives. The cleavage and polyadenylation are suppressed by the splicing-incompetent SDm5 mutant. The significances of the reductions by the MSD mutations were calculated by paired two-sample t-test. p-value is indicated. (**B**) Northern blotting of RNA from BHK-21 cells transfected with pCMVTas and the pGL3 derivatives detected with a probe encompassing nucleotides +1 to +250. Ratios of transcripts uncleaved/cleaved at the LTR polyadenylation site are shown below the Northern blot.

In order to confirm that suppression of the poly(A) site is independent of splicing but dependent on U1snRNP binding, a pGL3LTR reporter plasmid encoding an inactive splice donor mutant (SDm5) was constructed. This mutant encodes an ideal U1 binding site with the exception of the G/G dinucleotide. This dinucleotide was mutated to G/C, which has been shown to inhibit splicing (Figure 1A) [35]. BHK-21 cells transfected with pGL3SDm5 showed a slight decrease in luciferase activity of 23% compared to the wild-type (p = 0.01) (Figure 4A), likely due to the mismatch in U1snRNA-MSD binding (for luciferase data on SDm4 see S1). Nevertheless, the splicing-incompetent SDm5 suppressed 5´LTR polyadenylation compared to SDm2, showing that splicing is not required for suppression of polyadenylation.

To confirm these results, Northern blotting analysis using a probe encompassing the R region of the pGL3SDm5- and SDm5+p(A)m-derived transcripts was performed. RNAs were extracted using an miRNA isolation procedure (Figure 4B). The mutation SDm2 led to an increase in polyadenylation at the 5´LTR poly(A) site and a reduction of the read-through transcript (Figure 4B), which is in line with the results of the RPA. Consistent with the results of the luciferase assay, the Northern blot analysis revealed that SDm5 suppresses 5´LTR polyadenylation similar to the wild-type (compare lanes 1 and 3), indicating that splicing is not a prerequisite for poly(A) suppression. Nevertheless, transcript cleavage at the 5´LTR was not fully suppressed by SDm5, which contains 10 nucleotides complementary to the U1snRNA. A control transfection with inactivation of the 5´LTR poly(A) signal led to the expected polyadenylation at the vector’s SV40 polyadenylation signal (Figures 4B, lane 4). In addition, we confirmed by RT-PCR that SDm5+p(A)m supports polyadenylation at the SV40 polyadenylation site (Figure 2D, lane 7). In summary, we provide evidence that splicing is not a prerequisite for suppression of polyadenylation at the FV 5’LTR.

### Regulation of polyadenylation is promoter-independent

**Figure 5:**
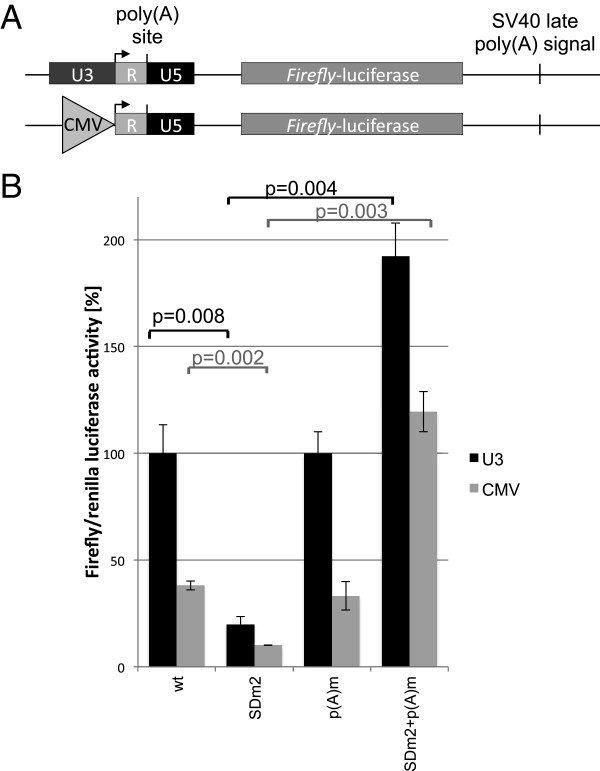
**Regulation of polyadenylation is promoter-independent.** Exchange of FV U3-promoter with the CMV-I.E. promoter has no influence on the regulation of polyadenylation. (**A**) Plasmid schemas are indicated above. (**B**) Bars represent the mean value of three independent transfections, and the error bars represent the standard deviations. The significance of the reduction by the SDm2 mutation or increase by SDm2+p(A)m was calculated by paired two-sample t-test. p-values are indicated.

Transcription, splicing, and poly(A) addition are coupled processes [1]. Since the HIV-1 U3 promoter and the CMV i.E. promoter recruit specific RNA-polymerase complexes II (Pol II) which display differences in both processivity and splicing [36], an analysis of the regulation of the FV polyadenylation concerning the promoter-dependency was desirable. The U3 promoter was excised from the pGL3LTR, -SDm2, and the respective poly(A) signal mutant constructs and replaced with the CMV-promoter fragment of pcHSRV2 [37] (Figure 5A). In these plasmids, the transcriptional start site of the constitutive CMV promoter is identical to the PFV transcriptional start site. Cellular luciferase activities after transfection with the U3 plasmid were more than 2-fold higher compared to cells transfected with the CMV plasmids, showing either a higher processivity of the recruited Pol II-complexes or a higher initiation rate at the FV LTR promoter. But the regulation of the polyadenylation was unaffected. The reduction of luciferase activity of the SDm2 transfected cells was in the same range as those transfected with the LTR promoter, and the additional poly(A) signal mutants displayed comparable increases in luciferase activities. This increase might be due to an inactive polyadenylation signal and to suppressed splicing by the SDm2 mutant. These results imply that the suppression of the 5’LTR polyadenylation of the 5’LTR is independent of the promoter.

### Regulation of polyadenylation at the 3′LTR

**Figure 6:**
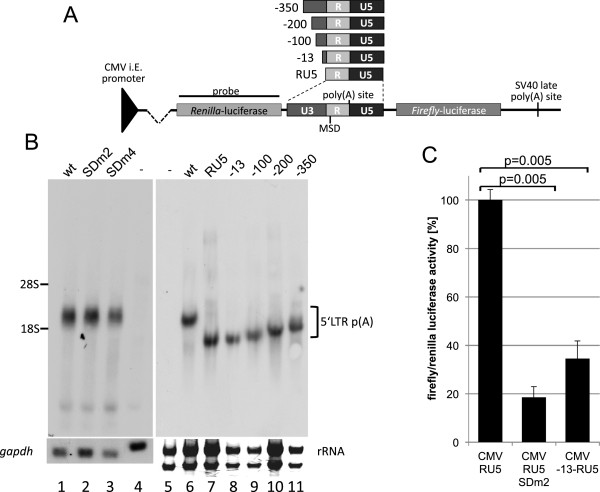
**Polyadenylation at the 3**′**LTR is splice**-**donor**-**independent,and 13 nucleotides from U3 are sufficient to promote polyadenylation.** (**A**) Schematic representation of the U3 deletion reporter plasmids used for analysis of polyadenylation at the 3′LTR and the position of the Northern blotting probe. (**B**) Northern blotting analysis of RNA from HEK 293T cells transfected with pHSRV13 and SDm2 or SDm4 derivatives. The RNAs were preferentially polyadenylated at the 3’LTR polyadenylation site. Neither SDm2 nor SDm4 influenced the poly(A) site selection. To analyse determinants of the poly(A) selection, the U3 region (777 nucleotides) was shortened to encompass nucleotides from −350, -200, -100, or −13 to +1. In addition, a plasmid encoding the RU5 region with a complete deletion of U3 was analysed. rRNA amounts are shown as loading control, since the RU5 and −13 signals migrate at the same height as GAPDH RNA. (**C**) Luciferase assay of cells transfected with CMV-RU5, CMVRU5-SDm2, and CMV -13RU5 showing that introns, exons, and coding sequences are not required for efficient polyadenylation. Assays were performed in triplicate, and error bars represent the standard deviation from the mean. The significances of the reductions by the MSD mutations were calculated by paired two-sample t-test. p-values are indicated.

In HIV-1, the MSD is located downstream of the 5′LTR. Therefore, polyadenylation at the 3′LTR, which lacks a downstream MSD, is not inhibited. In contrast, FVs have to prevent suppression of polyadenylation by the MSD at the 3′LTR because the R regions of both FV LTRs harbour an MSD. In order to determine the requirements for polyadenylation at the 3′end, we analysed whether the splice donor is essential for the regulation of polyadenylation. Either the wild-type LTR or the SDm2 mutants were inserted between *Renilla* and firefly luciferase genes in the pRL vector (Figure 6A). In addition, to find out whether a stronger MSD would suppress polyadenylation, we created a LTR mutant with 11 nucleotides of the MSD complementary to the U1snRNA (SDm4) by site-directed mutagenesis (Figure 1A) and inserted it into the 3′LTR reporter construct. The resulting constructs encode two poly(A) sites: 1) the FV LTR polyadenylation site (transcript size 2174 nts) and 2) the vector-derived SV40 late poly(A) site (transcript size 4125 nts) (Figure 6A). BHK-21 cells were transfected with the reporter constructs, and RNAs were analysed by Northern blotting using a probe encompassing *Renilla* luciferase (Figure 6B). These experiments were performed in the absence of Tas; however, further experiments showed that addition of Tas did not change the polyadenylation pattern nor did Tas activate the U3 promoter in these constructs, possibly indicating that Tas is unable to bind to 3′LTR sequences. The Northern blots showed that RNA was polyadenylated at the LTR and that the polyadenylation was independent of a functional splice donor (Figure 6B, lanes 2 and 3). Neither the weak splice donor SDm2 nor the strong SDm4 had any influence on polyadenylation site selection. These results were in striking contrast to all experiments with the LTR at the 5′position. To identify signals that support polyadenylation and render the splice donor non-relevant, we analysed the effects of U3 region deletions. The U3 region of pHSRV13 encompasses 777 nucleotides. Five additional reporter plasmids encompassing the RU5 region alone or RU5 and the U3 regions from −350, -200, -100, or −13 to +1 were constructed (Figure 6A). RNAs from BHK-21 cells transfected with these plasmids were analysed by Northern blotting (Figure 6B). All constructs showed a preferential polyadenylation at the LTR poly(A) site, indicating that the region from −13 to +1 and other upstream sequences relieve suppression of LTR polyadenylation. In addition, transcripts of the construct encoding only RU5 were polyadenylated at the LTR (Figure 6B, lane 7), supporting the hypothesis that U3 sequences or even upstream exons activate polyadenylation.

The RNA-region at the 3’LTR is preceded by the whole genomic pre-mRNA including pre-selected splice sites etc., whereas the RNA at the 5’poly(A) signal only encompasses the R-region. To further investigate whether 3′polyadenylation is influenced by upstream sequences, a simplified reporter was constructed by inserting nucleotides −13 to +1 into the pGL3-CMV-RU5 clones described above (Figure 5). In this construct, nucleotide −13 is positioned directly at the start site of the CMV promoter. Thus, the transcript is free of upstream coding regions, but encodes minimal sequences of U3. Transfection experiments showed that the additional 13 nucleotides of U3 caused a significant reduction in luciferase activity to 34% of the wild-type (p=0.005) (Figure 6C), which is comparable to the reduction seen with the SDm2 mutant (Figure 6C). This indicated that polyadenylation at the 3’LTR might have been activated by the 13 nucleotides of U3. In summary, these experiments show that U3 upstream sequences are able to activate polyadenylation at the 3’LTR.

### Differences in the RNA structure at the 5′ and 3′LTRs presumably regulate splice donor recognition

**Figure 7:**
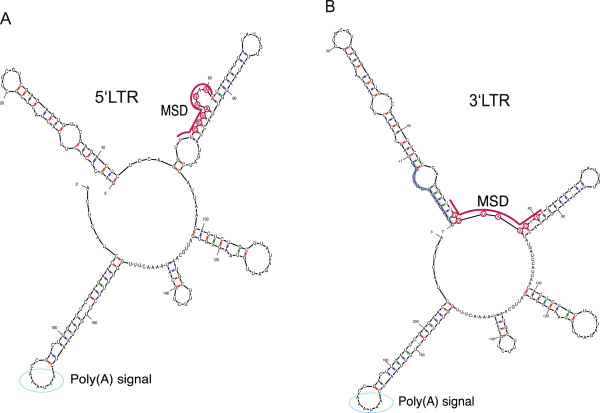
**SHAPE analysis of the RNA structure from (A)+1 to+198 and from (B)−13 to+198.** Secondary structure models of both RNAs developed using SHAPE constraints are shown. The positions of the MSD and polyadenylation signal are indicated and the region from −13 to −1 is shaded in violet. Nucleotides complementary to the U1snRNA are circled (red) and the position of the poly(A) signal is marked by blue circles.

To determine the differences in both polyadenylation and splice donor dependence at the 5′ and 3′LTRs, we analysed the RNA secondary structure of two RNA fragments, one representing the 3′LTR RNA (nucleotides −13 to +198) and one representing the 5′LTR RNA (nucleotides +1 to +198) by RNA SHAPE (Figure 7 and Additional file 1: Figure S2). The 3′ends of the two RNAs including the poly(A) signal and poly(A) site show identical secondary structural folds. However, we observed major differences at the 5′end of the two RNAs. Compared with the 5′LTR sequence (Figure 7A), the first stem loop of the 3′LTR is extended and the second stem loop is shortened (Figure 7B). The MSD is located between stem loops one and two in the 3′LTR, and only two nucleotides complementary to the U1snRNA are unpaired. The MSD of the 5′LTR is part of its extended second stem loop and forms a bulge. This leaves four U1snRNA-binding nucleotides unpaired. This structure is strikingly similar to the U1A-stem structure conserved in all mammals [38]. Additionally, we predicted the RNA secondary structures of the SDm1 and SDm2 mutants.

The single 5’LTR mutation of SDm1 completely disrupts the local RNA fold and modifies the RNA structure to a 3’LTR-like fold (Additional file 1: Figure S3). The 5’LTR SDm2 mutation repositions the bulge of the MSD by one nucleotide further upstream. This leads to substantial changes in the stem loop containing the MSD: a) the stem of the mutated RNA upstream of the bulge consists of six instead of five base paired nucleotides; b) the mutated base is no longer complementary to the U1snRNA; c) only two instead of three unpaired bases present in the bulge are complementary to the U1snRNA. In addition, structure prediction of a Renilla luciferase-R-U5 construct (Additional file 1: Figure S3C) representing the one used in Figure 6B (lane 7) shows disruption of 5’LTR MSD.

Taken together, our data provide evidence that adoption of deviating RNA structures in the 5’LTR MSD leads to premature polyadenylation.

## Discussion

Cellular polyadenylation must be tightly regulated as both premature polyadenylation, which would lead to non-functional transcripts, and non-polyadenylated transcripts, which would fail nuclear export, have to be prevented. Cellular genes and viral genomes often encode more than one functional poly(A) signal. Thus, cleavage and polyadenylation have to be repressed at these additional sites. Recent studies regarding the role of U1snRNA in the suppression of polyadenylation have revealed that functional inactivation of U1snRNPs results in the usage of cryptic poly(A) sites throughout the cellular genome [39]. This shows that the mechanisms of poly(A) suppression, described in this work for FVs, are used by the cell itself and that viruses likely adapted this strategy to achieve full-length RNA expression. We recently showed that a mechanism similar to that of suppression of polyadenylation at the 5′LTR of FVs is the origin of an inherited immunodeficiency syndrome [34]. A single nucleotide exchange (+23C>A) in the 3’UTR of the *p14*/*robld3* gene leads to the creation of a splice donor site. This upstream splice donor represses polyadenylation at the authentic *p14*/*robld3* poly(A) signal and is deleterious for *p14* mRNA biogenesis [34].

The FV 5′leader region and even deletions of the MSD have been characterized in several publications, but none addressed the influences of the 5′splice site on regulation of polyadenylation [25,29,30]. Here, we show that, in FVs, polyadenylation at the 5′LTR is controlled by the MSD, similar to the mechanism described for HIV-1 [16,18]. However, the R region of FVs unlike that of any other retroviruses contains an MSD upstream of the poly(A) site, and all elements required for polyadenylation are present in both LTRs. This contrasts sharply with the regulation of polyadenylation in HIV-1, in which the MSD is only present in the RNA at the 5′LTR, and the poly(A) site at the 3′LTR is therefore not inhibited. FVs solve this problem with a strong poly(A) site that is only partially inhibited by the MSD at the 5′LTR. This type of regulation ensures that viral full-length RNAs are quantitatively polyadenylated, even though some of these transcripts are cleaved at the 5′LTR. On the other hand, the incomplete poly(A) suppression lowers the amount of functional genomic RNAs produced in FVs and in FV-derived vector systems. If the amount of genomic RNA is a limiting factor, one should consider weakening the MSD and inactivating the poly(A) signal in the vector context, as this could prevent cryptic splicing and premature polyadenylation. In contrast, the HIV-1 MSD suppresses polyadenylation almost completely [9,17] and requires the R region for occlusion. As detailed mechanistic differences between polyadenylation in HIV-1 and MoMLV remain unknown, it is still unclear how HIV-1 achieves complete suppression. In MoMLV the MSD is attenuated by a combination of secondary structure and low complementarity to U1snRNA and thus, may not allow suppression of polyadenylation [23]. Possibly, unlike HIV-1, FVs may encode polyadenylation enhancer sequences, which would inhibit poly(A) occlusion. Our data imply that the deletion of the U5 or RU5 region in the 5`LTR results in higher transcript levels because by the deletion of the DSE the incomplete suppression of polyadenylation is abolished. Similar results were previously obtained with other FVs [40-42]. However, this phenomenon has always been explained by the existence of a transcriptional repressor of unknown identity.

In HIV-1, the promoter affects splicing [43]. When the HIV-1 U3 region was exchanged with the CMV promoter, the splicing pattern was changed. As splicing is thought to be associated with polyadenylation in general, and recognition of the splice donor is required for the suppression of polyadenylation in FVs, we analysed whether the exchange of the FV promoter would result in different regulation of poly(A) occlusion. However, this was not the case, which supports the model that U1snRNP binding to the MSD is responsible for poly(A) suppression. This was further substantiated by the finding that FV suppression of polyadenylation is dependent on the strength of the interaction between the MSD and the U1snRNA. In addition, by increasing the distance between the MSD and the poly(A) signal by 462 nucleotides, suppression of polyadenylation at LTR poly(A) site was relieved and additional inactivation of the MSD did not alter the polyadenylation efficiency (data not shown). In the case of HIV-1, this distance had to be increased by 1000 nucleotides to obtain a similar increase in polyadenylation frequency [18]. This leads us to conclude that the linear distance between the MSD and poly(A) signal is not the essential factor. Rather, the 3-dimensional RNA structure likely plays a key role. The RNA secondary folds of the 5’ and 3’LTR differ significantly in the MSD region and provide an explanation for the inability of the MSD at the 3’LTR to recognize U1snRNP (Figure 7). The structure of the MSD at the 5’LTR is strikingly similar to the structure of the U1snRNA binding site of U1A genes of mammals, which was shown to repress polyadenylation [38]. Minor changes in the 5’LTR, such as single mutations in the MSD (SDm1 and SDm2) or addition of 13 nucleotides at the 5’end of the R region (3’LTR), already cause a loss of the structural integrity of the U1snRNP recognition motif (Additional file 1: Figure S3). Therefore, prevention of polyadenylation at the 5’LTR is regulated by a highly sensitive and specific mechanism.

The data obtained by studying retroviral polyadenylation show that it takes place after U1snRNA binding to 5′splice sites. In HIV-1, for example, the poly(A) signal is located approximately 200 nucleotides upstream of the MSD. In addition, we can assume that splicing in retroviruses occurs after polyadenylation, because all retroviruses need a polyadenylated but unspliced genomic transcript to be exported from the nucleus. The regulation of FV polyadenylation at the 3′LTR seems to be more complex. In contrast to HIV-1 and MoMLV, FV-derived vectors show nearly complete read-through suppression at the 3′LTR [31]. Our experiments support the existence of a functional DSE in U5 although the G/U-rich region is nearly absent in FV LTR. The regulation of polyadenylation at the FV 3′LTR appears to be unique, as no other retrovirus encodes an MSD at the 3′LTR. We present evidence that FV polyadenylation is independent of U1snRNP binding to the 3′LTR, because neither a weak MSD (SDm2) nor a strong MSD (SDm4) changes the polyadenylation efficiency (Figure 6). The switch from polyadenylation suppression to almost complete polyadenylation is caused by upstream sequences.

## Conclusions

Foamy viruses have a unique R region structure. In this work, we characterized FV polyadenylation and showed that the RNA cleavage at the 5′LTR is suppressed by the MSD which resembles the mechanism used by HIV-1, although the order of the MSD and poly(A) site is exchanged. The specific mechanistic issue that FVs face is the regulation of polyadenylation at the 3′LTR, where suppression of cleavage and prevention of subsequent polyadenylation by the MSD must be abolished. We have collected evidence that this regulation might have been provided by an RNA structure that prevents U1snRNP binding.

## Methods

### Plasmids and mutants

The U1snRNA and pCMVTas expression plasmids and the pHSRV2, pHSRV2SDm1, and pHSRV13 proviral clones have been described previously [23,32,33]. pHSRV13 and pHSRV2 harbour identical proviral PFV full-length sequences but differ in their plasmid backbones. The U1snRNAm2 expression construct was generated by site-directed mutagenesis. A detailed description of primers and clones can be found in the Additional file 1. The LTR was amplified from pHSRV13 and inserted into a *Kpn*I/*Xho*I-digested pGL3-basic vector (Promega). Primers used for site-directed mutagenesis of the MSD or the poly(A) signal are listed in the Additional file 1. The LTR mutants were introduced into a subcloned *Eag*I/*Swa*I fragment of pHSRV13 and re-inserted into pHSRV13. The RPA probe was cloned into the pSC-B vector (Stratagene).

### Transient transfections

For luciferase assays, 1×10^4^ BHK-21 cells were maintained as previously described [44] and co-transfected with 20 ng *Renilla* luciferase expression plasmid (pCMV-RL, Promega), 40 ng pCMVTas, and 20 ng pGL-3LTR or derivatives, and TurboFect transfection reagent (Fermentas). The total amount of DNA was adjusted to 100 ng with pUC19 DNA (Invitrogen). Transfection efficiencies were normalized based on *Renilla* luciferase activity. All luciferase activity assays were performed independently in triplicate using the DualGlo-Kit (Promega) according to the manufacturer’s instructions. For the U1snRNA co-transfections, luciferase and pCMVTas expression constructs were used as described above. In addition, 100 ng of the respective U1snRNA expression constructs were co-transfected. For the U1snRNA competition assays with proviral constructs, BHK-21 cells were transfected with 1 μg of pHSRV13 or derivatives, 0.5 μg pCMVTas, and 2 μg U1snRNA or U1snRNAm2 expression construct.

### Northern blotting

For Northern blotting, 4×10^5^ BHK-21 cells were co-transfected with 1 μg pHSRV2 (or derivatives) or pGL3-LTR (or derivatives), 2 μg pCMVTas, and 0.5 μg peGFPC1. The preparation of total RNA was performed as previously described [44] or with miRNA purification kits according to the manufacturers’ instructions (Stratagene and Machery & Nagel). Five micrograms of RNAs were loaded onto a 1% agarose gel and transferred onto a Hybond-N+ membrane (Amersham) by capillary blotting. The blots were hybridized overnight at 60°C to a RU5-specific probe (activity >10^7^ cpm) that had been labelled by PCR using primers FV+1 and XhoILTRa (Additional file 1). The blots were re-hybridized to a human *GAPDH* gene (nucleotides 1011–1310) probe. Transcripts were quantified using the AIDA software package.

### Western blotting

BHK-21 cells were transfected as described above. Western blotting analyses of cellular lysates were performed two days after transfection using Gag-, integrase Tas-, and GAPDH-specific antisera [44].

### RNA synthesis and RPAs

Synthesis of all RNAs used in this study was performed with SP6 RNA Polymerase (Promega). To obtain ^32^P-labeled RNA, 0.74 MBq of ***α***[^32^P]-UTP (Hartmann Analytic) were included in the *in vitro* transcription assay. All RNA probes were purified by PAGE. The RPAs were performed using the RPAIII kit (Ambion) according to the manufacturer’s instructions.

### SHAPE

RNA synthesis, 5′*-*end labeling of the primer, selective 2′hydroxyl acetylation analyzed by primer extension (SHAPE), and sequencing reactions were performed as described by Hartl *et al*. with minor modifications [45]. RNAs spanning the region +1 to +198 or −13 to +198 were synthesized using the T3 MEGAscript kit (Applied Biosystems, Austin, TX) and subjected to SHAPE analysis [46-48]. SHAPE and sequencing reactions were performed with two different primers: one binding to the 3′*-*end of the RNAs (nucleotides +198 to +176, 5′-TGAGTAGGTTCTCGAATCAAGTC-3′; IBA, Göttingen, Germany) and the other binding to the central part of the RNAs (nucleotides 101 to 75, 5′ GTGTTAATGGATCATAGTAACATT ATA; IBA, Göttingen, Germany). The plasmid pHSRV13 was used as a template for sequencing reactions to assign the SHAPE reaction products. The program Mfold [49-51] was used to calculate RNA secondary structures. Nucleotides with SHAPE intensities higher than 0.3 were set to be unpaired. Low SHAPE intensities can be caused by either a nucleotide that is paired in the secondary, tertiary as well as quaternary RNA structure or by a high background signal. Therefore, we did not set constraints for paired nucleotides. For LTR mutants, only the regions of identical secondary structures in the wild*-*type 3′ and 5′LTR (nucleotides 99 to +198) were constrained to ensure the integrity of the folding topology of these regions, while the folding of nucleotides −13 to +98 was unconstrained.

## Abbreviations

DSE: Downstream element; FV: Foamy virus; HIV-1: Human immunodeficiency virus type 1; HTLV-I: Human T-Lymphotropic virus type I; LTR: Long terminal repeat; MMTV: Mouse mammary tumour virus; MoMLV: Moloney murine leukaemia virus; MSD: Major splice donor; poly(A) signal: Polyadenylation signal; poly(A) site: polyadenylation cleavage site; PFV: Prototype foamy virus; RSV: Rous sarcoma virus

## Competing interests

The authors declare that they have no competing interest.

## Authors’ contributions

E-MS, R M, M J H, JB, H W, D F, and J L performed the experiments. JB, Je B and BMW contributed to the data analysis and the design of the study. JB wrote the manuscript. All authors read and approved the final manuscript.

## Supplementary Material

Additional file 1 **Supplementary material. Figure S1.** SDm4 suppresses polyadenylation at the LTR poly(A) site efficiently. Luciferase assay of pGL3-LTR derivatives. BHK-21 cells were co-transfected with pGL3-LTR derivatives, a Tas expression plasmid and a CMV-driven Renilla-luciferase expression plasmid. Luciferase expression was measured 2d after transfection. The bars represent the mean of 3 independent experiments. The error bars indicate the calculated standard deviation. The experiment was repeated six times. **FigureS2.** Relative SHAPE intensities as a function of base position. The 5'LTR intensities are shown in white, the 3'LTR intensities in black bars. Bases with intensities higher than 0.3 were assumed not to be paired. The data are derived from a single SHAPE experiment. The experiment was repeated twice. The positions of the MSD and the polyadenylation signal are indicated. **Figure S3.** Calculated RNA structures of (**A**) SDm1, (**B**) SDm2, and (**C**) the Renilla luciferase-R-U5 construct using the constraints from the RNA SHAPE analyses (Figure 7). Click here for file

## References

[B1] Moore MJ Proudfoot NJ Pre-mRNA processing reaches back to transcription and ahead to translation Cell 2009 136 688 700 10.1016/j.cell.2009.02.001 19239889

[B2] Zhao J Hyman L Moore C Formation of mRNA 3' ends in eukaryotes: mechanism, regulation, and interrelationships with other steps in mRNA synthesis Microbiol Mol Biol Rev 1999 63 405 445 10357856 10.1128/mmbr.63.2.405-445.1999 PMC98971

[B3] Danckwardt S Hentze MW Kulozik AE 3' end mRNA processing: molecular mechanisms and implications for health and disease EMBO J 2008 27 482 498 10.1038/sj.emboj.7601932 18256699 PMC2241648

[B4] Chen F MacDonald CC Wilusz J Cleavage site determinants in the mammalian polyadenylation signal Nucleic Acids Res 1995 23 2614 2620 10.1093/nar/23.14.2614 7651822 PMC307082

[B5] Schrom EM Moschall R Schuch A Bodem J Regulation of retroviral polyadenylation Adv Virus Res 2013 85 1 24 23439022 10.1016/B978-0-12-408116-1.00001-X

[B6] Coffin JM Hughes SH Varmus HE 1997 Retroviruses Cold Spring Habor Laboratory Press 21433340

[B7] Tang H Kuhen KL Wong-Staal F Lentivirus replication and regulation Annu Rev Genet 1999 33 133 170 10.1146/annurev.genet.33.1.133 10690406

[B8] Ahmed YF Gilmartin GM Hanly SM Nevins JR Greene WC The HTLV-I Rex response element mediates a novel form of mRNA polyadenylation Cell 1991 64 727 737 10.1016/0092-8674(91)90502-P 1671761

[B9] Furger A Monks J Proudfoot NJ The retroviruses human immunodeficiency virus type 1 and Moloney murine leukemia virus adopt radically different strategies to regulate promoter-proximal polyadenylation J Virol 2001 75 11735 11746 10.1128/JVI.75.23.11735-11746.2001 11689654 PMC114759

[B10] Cleavinger PJ Kandala JC Guntaka RV The GT-rich sequence in the U5 region of Rous sarcoma virus long terminal repeat is required for transcription termination and 3' processing Folia Biol (Praha) 1997 43 153 160 9338122

[B11] Seiki M Hattori S Hirayama Y Yoshida M Human adult T-cell leukemia virus: complete nucleotide sequence of the provirus genome integrated in leukemia cell DNA Proc Natl Acad Sci U S A 1983 80 3618 3622 10.1073/pnas.80.12.3618 6304725 PMC394101

[B12] Millevoi S Vagner S Molecular mechanisms of eukaryotic pre-mRNA 3' end processing regulation Nucleic Acids Res 2009 38 2757 2774 20044349 10.1093/nar/gkp1176 PMC2874999

[B13] Rigo F Martinson HG Polyadenylation releases mRNA from RNA polymerase II in a process that is licensed by splicing RNA 2009 15 823 836 10.1261/rna.1409209 19304926 PMC2673064

[B14] Wang Z Burge CB Splicing regulation: from a parts list of regulatory elements to an integrated splicing code RNA 2008 14 802 813 10.1261/rna.876308 18369186 PMC2327353

[B15] Rigo F Martinson HG Functional coupling of last-intron splicing and 3'-end processing to transcription in vitro: the poly(A) signal couples to splicing before committing to cleavage Mol Cell Biol 2008 28 849 862 10.1128/MCB.01410-07 17967872 PMC2223410

[B16] Ashe MP Griffin P James W Proudfoot NJ Poly(A) site selection in the HIV-1 provirus: inhibition of promoter-proximal polyadenylation by the downstream major splice donor site Genes Development 1995 9 3008 3025 10.1101/gad.9.23.3008 7498796

[B17] Ashe MP Furger A Proudfoot NJ Stem-loop 1 of the U1 snRNP plays a critical role in the suppression of HIV-1 polyadenylation RNA 2000 6 170 177 10.1017/S1355838200991957 10688356 PMC1369903

[B18] Ashe MP Pearson LH Proudfoot NJ The HIV-1 5' LTR poly(A) site is inactivated by U1 snRNP interaction with the downstream major splice donor site EMBO J 1997 16 5752 5763 10.1093/emboj/16.18.5752 9312033 PMC1170206

[B19] Das AT Klaver B Berkhout B A hairpin structure in the R region of the human immunodeficiency virus type 1 RNA genome is instrumental in polyadenylation site selection J Virol 1999 73 81 91 9847310 10.1128/jvi.73.1.81-91.1999 PMC103811

[B20] Berkhout B HIV-1 as RNA evolution machine RNA Biol 2011 8 225 229 10.4161/rna.8.2.14801 21358278

[B21] Weichs An Der Glon C Monks J Proudfoot NJ Occlusion of the HIV poly(A) site Genes Development 1991 5 244 253 10.1101/gad.5.2.244 1995416

[B22] Gilmartin GM Fleming ES Oetjen J Activation of HIV-1 pre-mRNA 3' processing in vitro requires both an upstream element and TAR EMBO J 1992 11 4419 4428 1425577 10.1002/j.1460-2075.1992.tb05542.x PMC557016

[B23] Zychlinski D Erkelenz S Melhorn V Baum C Schaal H Bohne J Limited complementarity between U1 snRNA and a retroviral 5' splice site permits its attenuation via RNA secondary structure Nucleic Acids Res 2009 37 7429 7440 10.1093/nar/gkp694 19854941 PMC2794156

[B24] Löchelt M Foamy virus transactivation and gene expression Curr Top Microbiol Immunol 2003 277 27 61 10.1007/978-3-642-55701-9_2 12908767

[B25] Bodem J Löchelt M Delius H Flügel R Detection of subgenomic cDNAs and mapping of feline foamy virus mRNAs reveals complex patterns of transcription Virology 1998 244 417 426 10.1006/viro.1998.9113 9601510

[B26] Muranyi W Flügel RM Analysis of splicing patterns of human spumaretrovirus by polymerase chain reaction reveals complex RNA structures J Virol 1991 65 727 735 1846194 10.1128/jvi.65.2.727-735.1991 PMC239812

[B27] Desmet FO Hamroun D Lalande M Collod-Beroud G Claustres M Beroud C Human Splicing Finder: an online bioinformatics tool to predict splicing signals Nucleic Acids Res 2009 37 e67 10.1093/nar/gkp215 19339519 PMC2685110

[B28] HBond Score Web-Interface[http://www.uni-duesseldorf.de/rna/html/hbond_score.php]

[B29] Liu W Backes P Löchelt M Importance of the major splice donor and redefinition of cis-acting sequences of gutless feline foamy virus vectors Virology 2009 394 208 217 10.1016/j.virol.2009.08.028 19775717

[B30] Russell RA Zeng Y Erlwein O Cullen BR McClure MO The R region found in the human foamy virus long terminal repeat is critical for both Gag and Pol protein expression J Virol 2001 75 6817 6824 10.1128/JVI.75.15.6817-6824.2001 11435560 PMC114408

[B31] Hendrie PC Huo Y Stolitenko RB Russell DW A rapid and quantitative assay for measuring neighboring gene activation by vector proviruses Mol Ther 2008 16 534 540 10.1038/sj.mt.6300398 18209733

[B32] Heinkelein M Thurow J Dressler M Imrich H Neumann-Haefelin D McClure MO Rethwilm A Complex effects of deletions in the 5' untranslated region of primate foamy virus on viral gene expression and RNA packaging J Virol 2000 74 3141 3148 10.1128/JVI.74.7.3141-3148.2000 10708430 PMC111814

[B33] Löchelt M Zentgraf H Flügel RM Construction of an infectious DNA clone of the full-length human spumaretrovirus genome and mutagenesis of the bel 1 gene Virology 1991 184 43 54 10.1016/0042-6822(91)90820-2 1651600

[B34] Langemeier J Schrom EM Rabner A Zychlinski D Saborowski A Bohn G Mandel-Gutfreund Y Bodem J Klein C Bohne J U1 snRNP-mediated poly(A) site suppression is the molecular basis of a complex immunodeficiency EMBO J 2012 31 4035 4044 10.1038/emboj.2012.252 22968171 PMC3474926

[B35] Asang C Hauber I Schaal H Insights into the selective activation of alternatively used splice acceptors by the human immunodeficiency virus type-1 bidirectional splicing enhancer Nucleic Acids Res 2008 36 1450 1463 10.1093/nar/gkm1147 18203748 PMC2275126

[B36] Bohne J Wodrich H Kräusslich H Splicing of human immunodeficiency virus RNA is position-dependent suggesting sequential removal of introns from the 5' end Nucleic Acids Res 2005 33 825 837 10.1093/nar/gki185 15701754 PMC549389

[B37] Moebes A Enssle J Bieniasz PD Heinkelein M Lindemann D Bock M McClure MO Rethwilm A Human foamy virus reverse transcription that occurs late in the viral replication cycle J Virol 1997 71 7305 7311 9311807 10.1128/jvi.71.10.7305-7311.1997 PMC192074

[B38] Guan F Caratozzolo RM Goraczniak R Ho ES Gunderson SI A bipartite U1 site represses U1A expression by synergizing with PIE to inhibit nuclear polyadenylation RNA 2007 13 2129 2140 10.1261/rna.756707 17942741 PMC2080603

[B39] Kaida D Berg MG Younis I Kasim M Singh LN Wan L Dreyfuss G U1 snRNP protects pre-mRNAs from premature cleavage and polyadenylation Nature 2010 468 664 668 10.1038/nature09479 20881964 PMC2996489

[B40] Yang P Zemba M Aboud M Flügel RM Löchelt M Deletion analysis of both the long terminal repeat and the internal promoters of the human foamy virus Virus Genes 1997 15 17 23 10.1023/A:1007994527345 9354264

[B41] Mergia A Pratt-Lowe E Shaw KE Renshaw-Gegg LW Luciw PA cis-acting regulatory regions in the long terminal repeat of simian foamy virus type 1 J Virol 1992 66 251 257 1309244 10.1128/jvi.66.1.251-257.1992 PMC238282

[B42] Renne R Friedl E Schweizer M Fleps U Turek R Neumann-Haefelin D Genomic organization and expression of simian foamy virus type 3 (SFV-3) Virology 1992 186 597 608 10.1016/0042-6822(92)90026-L 1310187

[B43] Bohne J Kräusslich HG Mutation of the major 5' splice site renders a CMV-driven HIV-1 proviral clone Tat-dependent: connections between transcription and splicing FEBS Lett 2004 563 113 118 10.1016/S0014-5793(04)00277-7 15063733

[B44] Bodem J Schied T Gabriel R Rammling M Rethwilm A Foamy virus nuclear RNA export is distinct from that of other retroviruses J Virol 2011 85 2333 2341 10.1128/JVI.01518-10 21159877 PMC3067772

[B45] Hartl MJ Bodem J Jochheim F Rethwilm A Rösch P Wöhrl BM Regulation of Foamy Virus Protease Activity by Viral RNA - a Novel and Unique Mechanism Among Retroviruses J Virol 2011 85 4462 4469 10.1128/JVI.02211-10 21325405 PMC3126251

[B46] Merino EJ Wilkinson KA Coughlan JL Weeks KM RNA structure analysis at single nucleotide resolution by selective 2'-hydroxyl acylation and primer extension (SHAPE) J Am Chem Soc 2005 127 4223 4231 10.1021/ja043822v 15783204

[B47] Mortimer SA Weeks KM A fast-acting reagent for accurate analysis of RNA secondary and tertiary structure by SHAPE chemistry J Am Chem Soc 2007 129 4144 4145 10.1021/ja0704028 17367143

[B48] Wilkinson KA Merino EJ Weeks KM Selective 2'-hydroxyl acylation analyzed by primer extension (SHAPE): quantitative RNA structure analysis at single nucleotide resolution Nature protocols 2006 1 1610 1616 10.1038/nprot.2006.249 17406453

[B49] Zuker M Mfold web server for nucleic acid folding and hybridization prediction Nucleic Acids Res 2003 31 3406 3415 10.1093/nar/gkg595 12824337 PMC169194

[B50] Waugh A Gendron P Altman R Brown JW Case D Gautheret D Harvey SC Leontis N Westbrook J Westhof E RNAML: a standard syntax for exchanging RNA information RNA 2002 8 707 717 10.1017/S1355838202028017 12088144 PMC1370290

[B51] Zuker M Jacobson AB Using reliability information to annotate RNA secondary structures RNA 1998 4 669 679 10.1017/S1355838298980116 9622126 PMC1369649

