# Supplementary material for: U1snRNP-mediated suppression of polyadenylation in conjunction with the RNA structure controls poly (A) site selection in foamy viruses
Source: Retrovirology. 2013 May 29;10:55. doi: 10.1186/1742-4690-10-55 (PMC3694450; doi:10.1186/1742-4690-10-55)
Supplement: Additional file 1 — Supplementary material. Figure S1. SDm4 suppresses polyadenylation at the LTR poly(A) site efficiently. Luciferase assay of pGL3-LTR derivatives. BHK-21 cells were co-transfected with pGL3-LTR derivatives, a Tas expression plasmid and a CMV-driven Renilla-luciferase expression plasmid. Luciferase expression was measured 2d after transfection. The bars represent the mean of 3 independent experiments. The error bars indicate the calculated standard deviation. The experiment was repeated six times. FigureS2. Relative SHAPE intensities as a function of base position. The 5'LTR intensities are shown in white, the 3'LTR intensities in black bars. Bases with intensities higher than 0.3 were assumed not to be paired. The data are derived from a single SHAPE experiment. The experiment was repeated twice. The positions of the MSD and the polyadenylation signal are indicated. Figure S3. Calculated RNA structures of (A) SDm1, (B) SDm2, and (C) the Renilla luciferase-R-U5 construct using the constraints from the RNA SHAPE analyses (Figure 7). [file 1742-4690-10-55-S1.pdf]

Supplementary materials

Since the data on the location of PFV polyadenylation site and on the sequences of the R-region were unclear, we sought to confirm both the polyadenylation site and R-region of the proviral plasmid pHSRV. The R-region was sequenced from several plasmids including an original stock of pHRSV13 obtained from M. Löchelt (DKFZ, Heidelberg). The sequences showed an additional cg-dinucleotide at position +6. This defines the position of the MSD at nucleotide +53.

In order to determine the polyadenylation site of PFV a RT-reaction was performed using RNA from PFV infected BHK cells and Oligo d(T) as anti-sense primer. The cDNA were amplified by PCR with Oligo d(T) and the primer +1. The PCR amplicon was sequenced and the polyadenylation site determined at position +193 in the 5' LTR.

The reporter constructs were cloned by amplifying the PFV LTR from pHSRV13 by PCR with the primers 5'LTR *Kpn*I and 3'LTR *Xho*I. The PCR product was digested with *Kpn*I/*Xho*I and cloned into the pre-digested pGL3 vector.

The MSD or poly(A) signal mutants were constructed by site directed mutagenesis using the 5'LTR KpnIs, 3'LTR XhoIa, p(A)mnt(a/s), and SD2/4(a/s) primers.

The U1snRNA mutant was cloned by PCR mutagenesis using a reverse primer including a *BamH*I restriction site (rv: 5'-CGC GGA TCC TCC ACT GTA GGA TTA AC-3') and a forward primer containing the U1 mutation flanked by a *Bgl*II restriction site (fw: 5'-GCC CGA AGA TCT CAT A**C**T GAC CTG-3'). The PCR product was cloned as a *BamH*I/*BglI*I fragment into the pUC19 U1wt plasmid (kindly provided by A. Weiner, Seattle, USA).

The U3 promoter was exchanged with the CMV i.E. promoter by digesting pcHSRV2 with *Nru*I, followed by a fill-in reaction using Klenow enzyme and a second digest with *Sac*I. The CMV promoter fragment was ligated into the *Acc65*I/blunt & *Sac*I digested pGL3-PFVLTR vector.

The nucleotide sequences of all constructs were determined with the 3'LTR XhoIa primer.

Primers

3'LTR XhoIa	TTTCTCGAGTGTCATGGAATTTTGTATATTGATTATC
5'LTR KpnIs	AAAGGTACCTGTGGTGGAATGCCACTAGAAACTAGG
+1	GAGCTCTTCACTACTCGCTGCGTCG
SD2s	TCTCAAGGTTTGGTCAGAAATATTTTATATG
SD2 as	AATATTTCTGACCAAACCTGGAGAGTCTCG
SD4s	TCTCCAGGTCAGGTAAGTATTATTTTATATTG
SD4 as	AAATAATACTTACCTGACCTGGAGAGTCTCGTAC
P(A)mnt s	GTTAATCTAATAACCGACTTGATTCGAGAACC
P(A)mnt as	AATCAAGTCGGTTATTAGATTAACAAGAAAACC

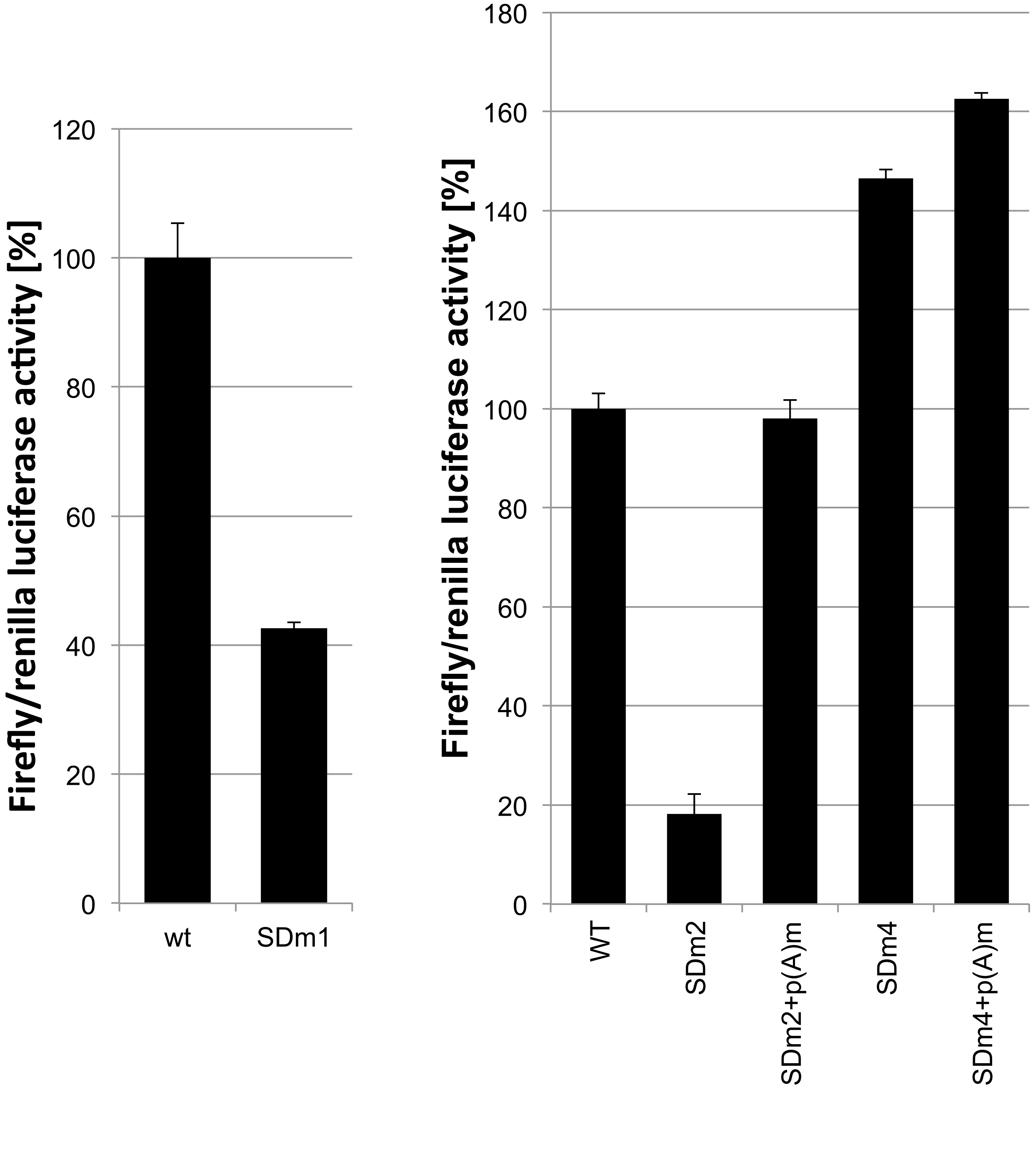

Figure S1: SDm4 suppresses polyadenylation at the LTR poly(A) site efficiently. Luciferase assay of pGL3-LTR derivatives. BHK-21 cells were co-transfected with pGL3-LTR derivatives, a Tas expression plasmid and a CMV-driven Renilla-luciferase expression plasmid. Luciferase expression was measured 2d after transfection. The bars represent the mean of 3 independent experiments. The error-bars indicated the calculated standard deviation. The experiment was repeated six times.

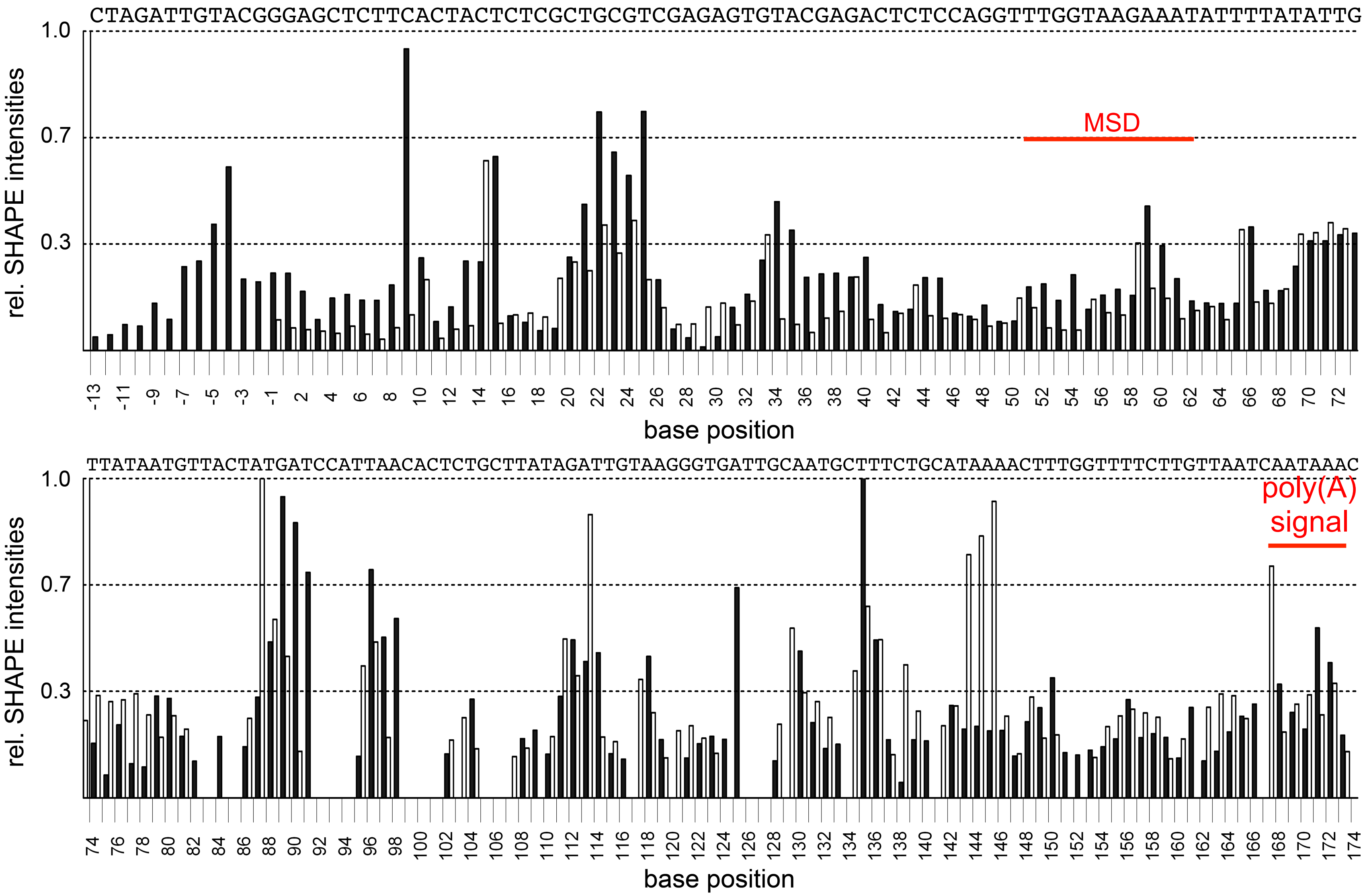

Figure S2: Relative SHAPE intensities as a function of base position. The 5'LTR intensities are shown in white, the 3'LTR intensities in black bars. Bases with intensities higher than 0.3 were assumed not to be paired. The data are derived from a single SHAPE experiment. The experiment was repeated twice. The positions of the MSD and polyadenylation signal are indicated.

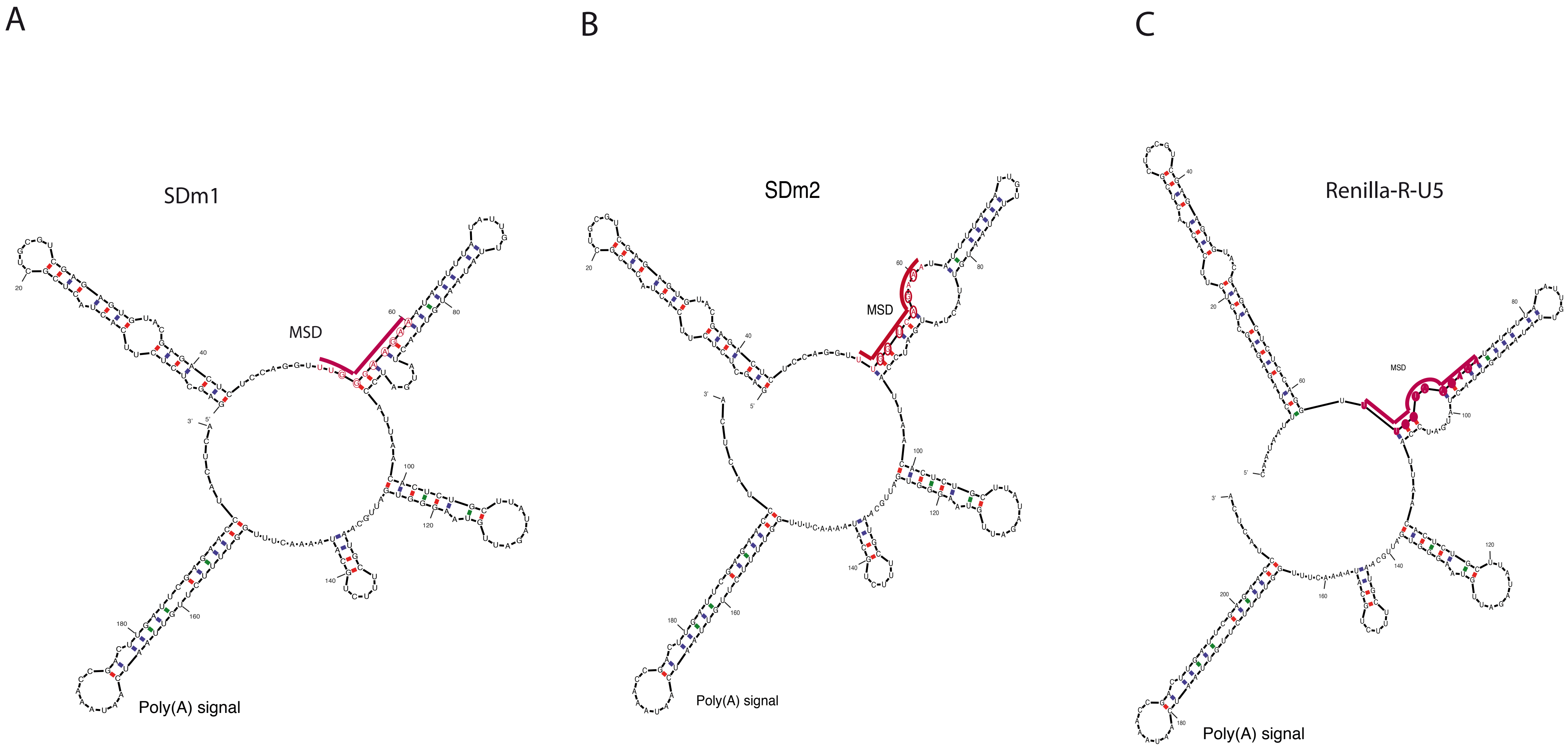

Figure S3: Calculated RNA structures of (A) SDm1, (B) SDm2, and (C) the Renilla luciferase-R-U5 construct using the constrains from the RNA SHAPE analyses (Figure 7)
